# Caspase-3, myogenic transcription factors and cell cycle inhibitors are regulated by leukemia inhibitory factor to mediate inhibition of myogenic differentiation

**DOI:** 10.1186/2044-5040-1-17

**Published:** 2011-04-07

**Authors:** Liam C Hunt, Aradhana Upadhyay, Jalal A Jazayeri, Elizabeth M Tudor, Jason D White

**Affiliations:** 1 Faculty of Veterinary Science, University of Melbourne, Flemington Road, Parkville, Victoria 3010, Australia; 2 Murdoch Childrens Research Institute, Royal Children's Hospital, Flemington Road, Parkville, Victoria 3052, Australia; 3 Medicinal Chemistry and Drug Action, Monash Institute of Pharmaceutical Sciences, Monash University (Parkville Campus), 381 Royal Parade, Parkville, Victoria 3052, Melbourne, Australia

## Abstract

**Background:**

Leukemia inhibitory factor (LIF) is known to inhibit myogenic differentiation as well as to inhibit apoptosis and caspase-3 activation in non-differentiating myoblasts. In addition caspase-3 activity is required for myogenic differentiation. Therefore the aim of this study was to further investigate mechanisms of the differentiation suppressing effect of LIF in particular the possibility of a caspase-3 mediated inhibition of differentiation.

**Results:**

LIF dependent inhibition of differentiation appeared to involve several mechanisms. Differentiating myoblasts that were exposed to LIF displayed increased transcripts for c-fos. Transcripts for the cell cycle inhibitor p21 as well as muscle regulatory factors myoD and myogenin were decreased with LIF exposure. However, LIF did not directly induce a proliferative effect under differentiation conditions, but did prevent the proportion of myoblasts that were proliferating from decreasing as differentiation proceeded. LIF stimulation decreased the percentage of cells positive for active caspase-3 occurring during differentiation. Both the effect of LIF inhibiting caspase-3 activation and differentiation appeared dependent on mitogen activated protein kinase and extracellular signal regulated kinase kinase (MEK) signalling. The role of LIF in myogenic differentiation was further refined to demonstrate that myoblasts are unlikely to secrete LIF endogenously.

**Conclusions:**

Altogether this study provides a more comprehensive view of the role of LIF in myogenic differentiation including LIF and receptor regulation in myoblasts and myotubes, mechanisms of inhibition of differentiation and the link between caspase-3 activation, apoptosis and myogenic differentiation.

## Background

Myogenic differentiation is a critical process for the development and homeostasis of muscle tissue. Myogenesis, the formation of muscle cell syncytia, occurs during embryonic development and in cases of muscle injury. When myofibers are damaged by stimuli such as mechanical stress, or loss of neurotrophic support, they regenerate by activation and proliferation of the normally quiescent resident satellite cell population [1]. Proliferating satellite cells, termed myoblasts, subsequently differentiate and fuse to create myotubes which can mature into functional myofibers. These mono-nucleated muscle progenitor cells differentiate by inducing the transcriptional activity of basic-helix-loop-helix transcription factors such as myoD and myogenin [2,3]. Commonly called muscle regulator factors (MRFs), these transcription factors initiate irreversible cell cycle arrest via increasing expression of p21 [4], which subsequently inhibits cyclin dependent kinase-2 (cdk-2) activity preventing cell cycle progression [5]. Myoblast cell membranes then fuse to create multinucleated syncytial cells known as myotubes [6]. Whilst these post-mitotic syncytia are resistant to apoptosis, up until the point of increased expression of cdk inhibitors such as p21 during differentiation, myoblasts are susceptible to apoptosis [7]. The process of myogenic differentiation is associated with typical apoptotic signalling such as caspase-3 activation, not only coinciding with differentiation but necessary for progression of differentiation [8]. Although differentiation associated apoptotic signalling may contribute positively to myogenic differentiation, it may also negatively lead to erroneous cell death [9]. Various proteins including growth factors and cytokines can regulate myogenic differentiation. One such cytokine, which shows increased expression in injured muscle undergoing myogenesis, is leukemia inhibitory factor (LIF) [10].

LIF conforms to the gp130 signalling of interleukin-6 family cytokines and is shown to inhibit differentiation of myoblasts [11]. LIF binds to a heterodimer of gp130 and the LIF receptor (LIFR) [12], which can lead to activation of a number of signalling pathways. These include signal transducer and activator of transcription 3 (STAT3), phosphotidylinositol-3 kinase (PI3K) and mitogen activated protein kinase kinase (MEK) [13]. LIF also inhibits caspase-3 activation and DNA fragmentation of myoblasts caused by induction of apoptosis with staurosporine [14]. Inhibition of myoblast differentiation by LIF is shown to be dependent on MEK signalling and independent of STAT3, while inhibition of staurosporine induced apoptosis was PI3K dependent [14]. Given the association between myogenic differentiation and apoptotic signalling and the involvement of LIF in both these processes separately we thought it prudent to determine if LIF influences differentiation-associated apoptotic signalling and to examine the mechanisms responsible for inhibition of myogenic differentiation by LIF.

LIF has been shown to play a role and display increased expression in regenerating muscle tissue [10,15]. This is comprised of numerous cell types including but not limited to neurons, fibroblasts and macrophages as well as myoblasts. However little is known about the expression and function of endogenous LIF during myoblast differentiation alone despite inhibition by exogenous LIF. We therefore set out to examine the regulation and function of endogenous LIF by myoblasts as well as to investigate mechanisms of inhibition of differentiation by LIF. Herein we describe the inhibition of myogenic differentiation of myoblasts by LIF and show that this effect is mediated by inhibition of caspase-3 activation, down-regulation of myogenic transcription factors myoD and myogenin and cell cycle inhibitor p21 whilst up-regulating the immediate early gene c-fos.

## Results

### Exogenous LIF delays myoblast differentiation and myotube formation

**Figure 1:**
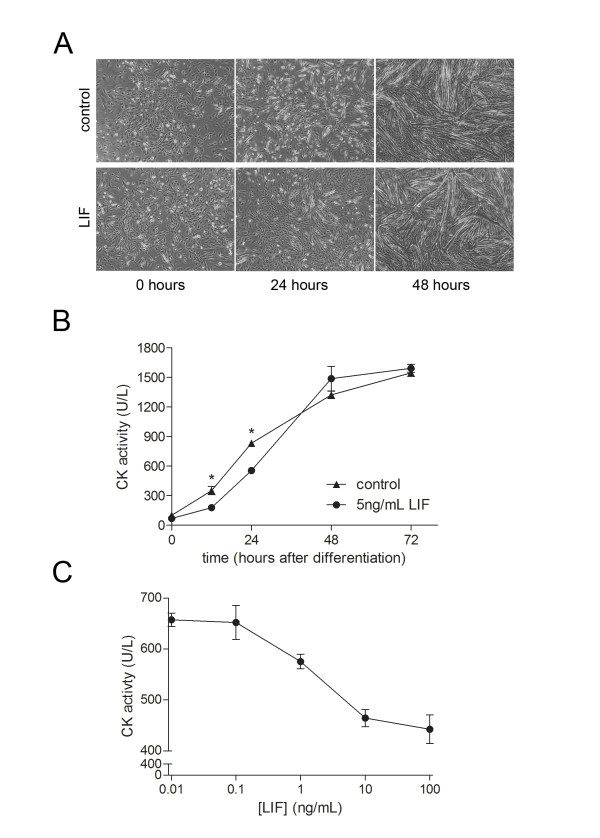
**LIF inhibits myotube formation and differentiation measured by creatine kinase activity**. (A) Phase contrast images of myoblast cultures either with or without 10 ng/mL LIF treatment at 0, 24 and 48 hours after induction of differentiation showed qualitatively less myotubes present at 24 hours of differentiation with LIF treatment. (B) Creatine kinase activity of myogenic cultures is decreased with 10 ng/mL LIF treatment at 12 and 24 but not 48 and 72 hours following the induction of differentiation. Asterisk (*) indicates *P *< 0.05 for LIF-treated compared to control by Student's t-test at that time (n = 4). (C) At 24 hours of differentiation, inhibition of creatine kinase activity with increasing concentrations of LIF, up to a maximum effect with 10 ng/mL LIF, was observed (n = 4).

A visual comparison of cultures incubated with 10 ng/mL LIF compared to untreated controls showed that 24 hours after differentiation was induced there appeared to be qualitatively less myotubes present in the LIF-treated compared to control cultures (Figure 1A). From 48 hours and onwards myotube formation appeared to have reached a maximum and there was no discernible difference in the size, amount or density of myotubes present with LIF treatment. Creatine kinase (CK) enzymatic activity increases over time as myoblasts differentiate and persists in fused myotubes [16]. Thus we used CK activity as a measure of the effect of exogenous LIF administration on C2C12 myoblasts differentiation. CK activity increased in control cultures induced to differentiate up to a maximum of 48 hours similar to myotube formation visualised in Figure 1A. LIF treatment significantly decreased this compared to control at the time points of 12 and 24 hours prior but not 48 hours or later (Figure 1B). This decrease at 24 hours (t = 24) caused by LIF treatment was dose dependent with concentrations of LIF 10 ng/mL and greater inducing a maximum inhibitory effect representing a 30% reduction from control values (Figure 1C). Overall these results indicate that LIF may slow the rate of differentiation of myoblasts, but does not prevent myoblasts from reaching the same endpoint of differentiation and myotube formation.

### LIF treatment inhibits differentiation associated apoptotic signalling and DNA fragmentation

**Figure 2:**
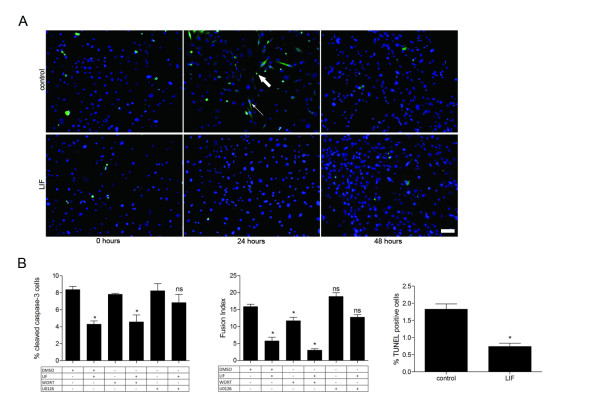
**LIF inhibits activation of caspase-3 and DNA fragmentation during differentiation**. (A) Representative immunofluorescent images of 10 ng/mL LIF-treated and control cultures labelled for cleaved caspase-3 (green) and DNA with DAPI (blue). A cleaved caspase-3 positive myoblast with more typical apoptotic morphology with condensed chromatin and small cytoplasmic space is indicated with a thick arrow. A cleaved caspase-3 positive myoblast with normal fusiform morphology is indicated with a thin arrow. Scale bar is 100 μm. (B) Cultures differentiated for 24 hours and immunofluorescently stained for cleaved caspase-3 and MYHC simultaneously were counted and the percentages of cleaved caspase-3 positive nuclei (of total nuclei) and fusion index (percentage of nuclei contained within MYHC positive syncytia) represented graphically. -/+ indicates either the absence or presence of LIF (10 ng/mL), wortmannin (WORT; 100 nM), U0126 (10 μM) or DMSO (0.1% (v/v) in media as the vehicle for wortmannin and U0126) treatment. Asterisk (*) indicates *P *< 0.05 and ns indicates *P *> 0.05 with one-way ANOVA and Dunnet's post-hoc test for all treatments compared to control (n = 4). (C) After 24 hours of differentiation cultures were TUNEL stained and the percentage of total cells positive for TUNEL represented graphically. Asterisk (*) indicates *P *< 0.05 for LIF-treated compared to control with Student's t-test (n = 4).

The number of myoblasts positive for the active cleaved caspase-3 (as a proportion of total cells) increased following induction of differentiation. Not all active caspase-3 positive myoblasts appeared apoptotic in morphology, suggesting that caspase-3 activation may not represent signalling towards apoptotic cell death but rather may relate to signalling of myogenic differentiation. Caspase-3 activation was greatest 24 hours after differentiation and had subsided to basal levels by 48 hours when maximum differentiation and myotube formation was observed (Figure 2A). Percentages of cells positive for active caspase-3 were lower in LIF-treated cultures at 24 hours but not at 48 hours, temporally consistent with when the effect of LIF on myotube formation and differentiation was observed (Figure 1). Pharmacological inhibitors of MEK and PI3K signalling pathways along with dual staining of active caspase-3 and myosin heavy chain (MYHC, a marker of differentiated myoblasts) was used at this 24 hour time point (t = 24) to examine the relationship between LIF, caspase-3 activation, differentiation and the signalling pathways involved. At this 24 hour time point LIF treatment alone reduced the percentage of cells positive for active caspase-3 by 50% and reduced the fusion index (the number of nuclei contained within MYHC positive syncytia as a percentage of total) by 64% compared to control (Figure 2B). The effects of LIF on caspase-3 activation and fusion index were not prevented by inhibition of the PI3K pathway with wortmannin. In fact, wortmannin alone significantly decreased the fusion index. Inhibition of MEK signalling with U0126 in the presence of LIF restored caspase-3 activation and fusion index to levels that were not significantly different from control, suggesting that the caspase-3 activation and differentiation are intertwined and are both inhibited by LIF through activation of MEK signalling. TUNEL positive cells were observed, with the highest levels (2% of total) 24 hours after induction of differentiation. This was consistent with the time course of caspase-3 activation. At this time point LIF treatment also decreased the percentage of TUNEL positive cells by 60% compared to control (Figure 2C).

### Caspase-3 inhibition blocks myogenic differentiation but is not additive with LIF

**Figure 3:**
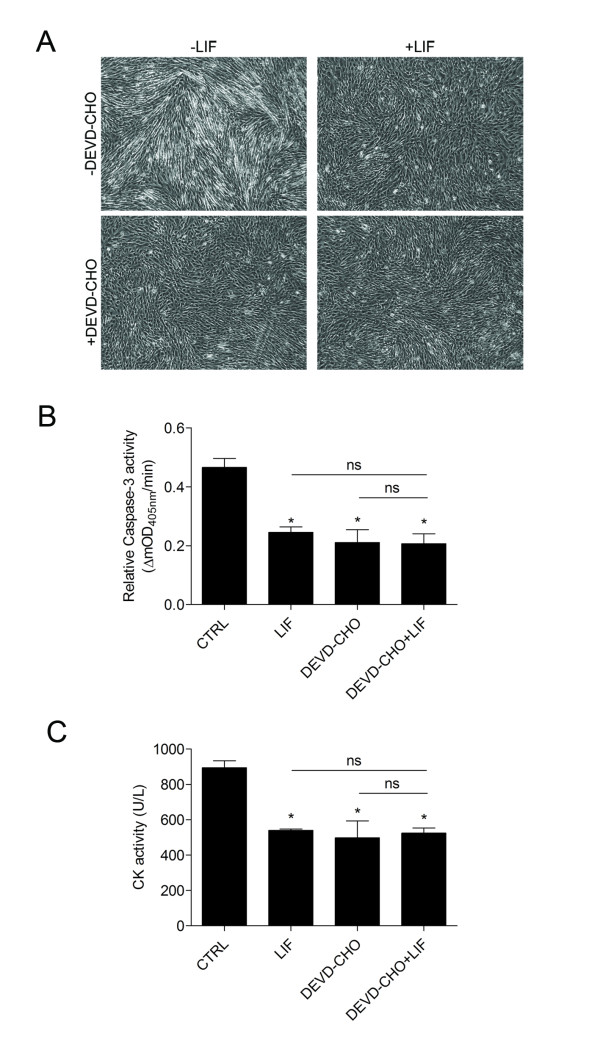
**Caspase-3 inhibition and LIF both inhibit myogenic differentiation and caspase-3 activity**. (A) Phase contrast images of myoblast cultures either with or without 10 ng/mL LIF or 100 μM Ac-DEVD-CHO (DEVD-CHO) treatment after 24 hours of differentiation showed qualitatively less myotubes present with LIF and Ac-DEVD-CHO and the combination of both compared to control. (B) Colormetric assay for caspase-3 proteolytic activity in cultures lysed 24 hours after differentiation. Caspase-3 activity is reduced with LIF, Ac-DEVD-CHO and the combination of LIF and Ac-DEVD-CHO. (C) Creatine kinase activity from the same lysates is also decreased with LIF, Ac-DEVD-CHO and the combination of LIF and Ac-DEVD-CHO indicating that inhibition of differentiation correlates with inhibition of caspase-3 proteolytic activity. Asterisk (*) indicates *P *< 0.05 and ns indicates *P *> 0.05 with one-way ANOVA and Bonferroni's post-hoc test for all treatments compared to each other (n = 4).

A small peptide Ac-DEVD-CHO, which mimics the substrate recognition sequence for caspase-3, reversibly binds to the active site of cleaved caspase-3 and inhibits the proteolytic activity, was utilized to discern whether caspase-3 inhibition blocks myogenic differentiation to the same degree as LIF and if inhibition of LIF and caspase-3 would be additive. Myoblasts were incubated with or without 10 ng/mL LIF and 100 μM Ac-DEVD-CHO prior to differentiation as described and after 24 hours of culture in differentiation media lysed for a colorimetric assay of caspase-3 proteolytic activity and CK activity. Assay of undifferentiated controls indicated that caspase-3 activity increased as differentiation proceeded similar to the immunocytochemical detection method (results not shown). Phase contrast images of differentiated cultures suggested that both LIF and Ac-DEVD-CHO treatment prevented formation of myotubes compared to control and the combination of LIF and Ac-DEVD-CHO also prevented formation of myotubes (Figure 3A). Caspase-3 proteolytic activity was significantly reduced with LIF treatment by a similar proportion to that observed with immunocytochemical detection of cleaved caspase-3 (Figure 3B). As expected Ac-DEVD-CHO inhibited caspase-3 activity, but interestingly the combination of LIF and Ac-DEVD-CHO did not reduce caspase-3 activity more than LIF or Ac-DEVD-CHO alone and therefore did not appear to be additive. CK activity was also measured from these lysates and paralleled the change in caspase-3 activity (Figure 3C). LIF and Ac-DEVD-CHO alone decreased CK activity in the 24-hour differentiated cultures as did the combined treatment. The combination of LIF and Ac-DEVD-CHO also did not appear to have an additive effect of reducing CK activity. These results are consistent with the immunocytochemical analysis that LIF inhibits caspase-3 activity during myogenic differentiation, which parallels a reduction in measures of differentiation.

### LIF alters transcripts of myogenic regulators and prevents decreases in proliferation caused by induction of differentiation

**Figure 4:**
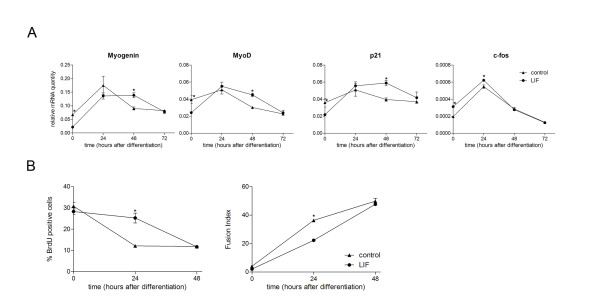
**LIF alters transcripts of genes relevant to myogenic differentiation and cell cycle progression and prevents decreases in proliferation due to differentiation**. (A) Real-time qPCR to determine relative mRNA quantity of the genes for myogenin, myoD, p21 and c-fos comparing LIF-treated to control over 72 hours of differentiation. Asterisk (*) indicates *P *< 0.05 by pair wise fixed reallocation randomization tests with REST for LIF- treated compared to control at that specific time point (n = 4). (B) Dual immunofluorescently labelled myogenic cultures for incorporated BrdU and desmin from 0 to 48 hours after differentiation were counted and represented as a percentage of total cells positive for incorporated BrdU and fusion index (percentage of nuclei contained within desmin positive syncytia). Asterisk (*) indicates *P *< 0.05 with Student's t-test for LIF compared to control at that time point (n = 4).

Analysis using real-time qPCR of transcripts important for myoblast differentiation showed an overall trend for decreased levels of positive regulators of myogenesis shortly after LIF treatment (Figure 4A). Transcripts for the MRFs myogenin and myoD were decreased at t = 0, which corresponds to 24 hours of LIF exposure and before differentiation is induced, suggesting that LIF decreases these messenger RNAs in the proliferating myoblasts. There was no significant difference at t = 24 with LIF treatment (*P *> 0.05) for both myogenin and myoD and at t = 48 these transcripts were significantly up-regulated in the LIF-treated group (*P *< 0.05 for myogenin and myoD). The cell cycle inhibitor p21 also followed the same trend as myogenin and myoD with significant decreases at t = 0, no significant difference at t = 24 and significantly increased at t = 48 with LIF treatment. The overall trend for these positive regulators of myogenesis, with a rightward shift on the time axis, suggests that LIF treatment causes a temporary down-regulation of these transcripts which is later negated, with no significant difference at t = 24, and even compensated for as differentiation proceeded (significantly up-regulated at t = 48). Expression of c-fos, which is an immediate early gene that can negatively regulate differentiation of myoblasts through repression of myogenin and myoD [17,18], was significantly increased with LIF treatment at t = 0 and t = 24, but not significantly different at t = 48 and t = 72. To further investigate whether inhibition of myogenic differentiation could be due to LIF inducing proliferation, as has been suggested previously [11,19], we examined proliferation and differentiation simultaneously following LIF treatment via immunofluoresence utilizing bromodeoxyuridine (BrdU) incorporation in DNA synthesizing cells and desmin to derive a fusion index of differentiated cells (Figure 4B). BrdU incorporation and thus DNA synthesis had occurred in 30% of cells before differentiation was induced (t = 0) which decreased to 12% 24 hours after differentiation had been induced (t = 24) and remained at that level until differentiation had reached a maximum (t = 48) in the control group. LIF exposure for 24 hours (t = 0) did not directly increase the percentage of myoblasts positive for BrdU incorporation suggesting LIF does not directly induce proliferation. This is more consistent with recent studies [14,20]. However the decreases in BrdU positive cells that occurred after 24 hours of differentiation in the control group did not occur with LIF treatment until t = 48 when LIF-treated cultures showed the same degree of differentiation as control cultures. Overall these results may indicate that LIF inhibits cell cycle withdrawal via transient up-regulation of c-fos and down-regulation of the cdk inhibitor p21 which maintains myoblasts in a proliferative state without directly inducing proliferation and therefore inhibiting differentiation temporarily.

### LIF is found in growth media and regulates differentiation and differentiation associated apoptosis

**Figure 5:**
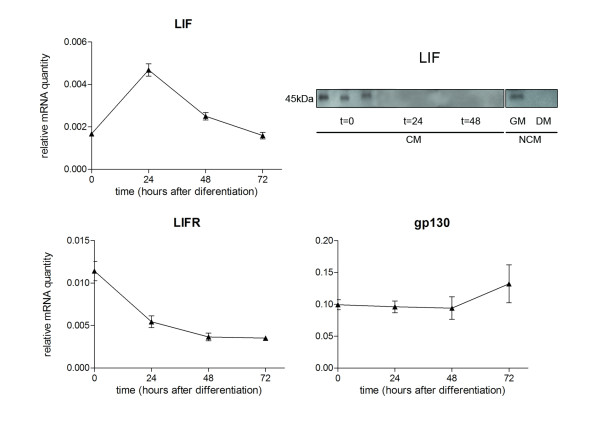
**Expression of endogenous transcripts for LIF and receptor components and LIF protein in conditioned media during differentiation**. Real-time qPCR for transcripts of LIF, LIFR and gp130 showed increased LIF with a peak 24 hours after differentiation, decreased LIFR following differentiation and relatively consistent levels of gp130 throughout differentiation. Western immunoblot showed the presence of 45 kDa glycoslyated LIF in the conditioned media (CM) comprised of growth media at t = 0 but not conditioned media comprised of differentiation media t = 24 or t = 48 hours. The same 45 kDa band was observed in growth media (GM) but not differentiation media (DM) that was not cultured with cells, that is non-conditioned media (NCM).

Although it is well established that myoblasts are sensitive to LIF it is not well known whether myoblasts may be responsible for the production and secretion of LIF as an autocrine factor. Therefore we examined the regulation of endogenous LIF during myogenesis *in vitro*. LIF transcripts, as measured by qPCR, were detected in mRNA isolated from undifferentiated myoblasts as well as differentiated cultures, with the greatest level of transcript present at t = 24 (Figure 5), suggesting that LIF may be produced in greatest quantity by myoblasts that are in the process of differentiating. Somewhat paradoxically, immunoreactivity by western immunoblot for a 45 kDa protein, representing glycosylated LIF, could only be detected in conditioned media (CM) taken from proliferating myoblasts (t = 0) and not after induction of differentiation when transcripts were greatest. However, LIF was detected in the growth media (GM) but not differentiation media (DM) of non-conditioned media (NCM) that is fresh media that was not cultured with cells. As t = 0 CM consisted of cultured GM while t = 24 and t = 48 CM consisted of cultured DM, the LIF protein detected in t = 0 CM is likely derived from the GM itself and so would obscure detection of any endogenously produced LIF. However we could not detect any LIF at t = 24 when transcripts were greatest suggesting that the level of endogenously produced LIF was too low to be detected. The only difference between GM and DM is the type and percentage of serum present, GM contains 10% fetal bovine serum whereas DM contains 2% normal horse serum. Therefore the LIF detected in GM and t = 0 CM is quite likely to be bovine LIF present in the fetal bovine serum.

**Figure 6:**
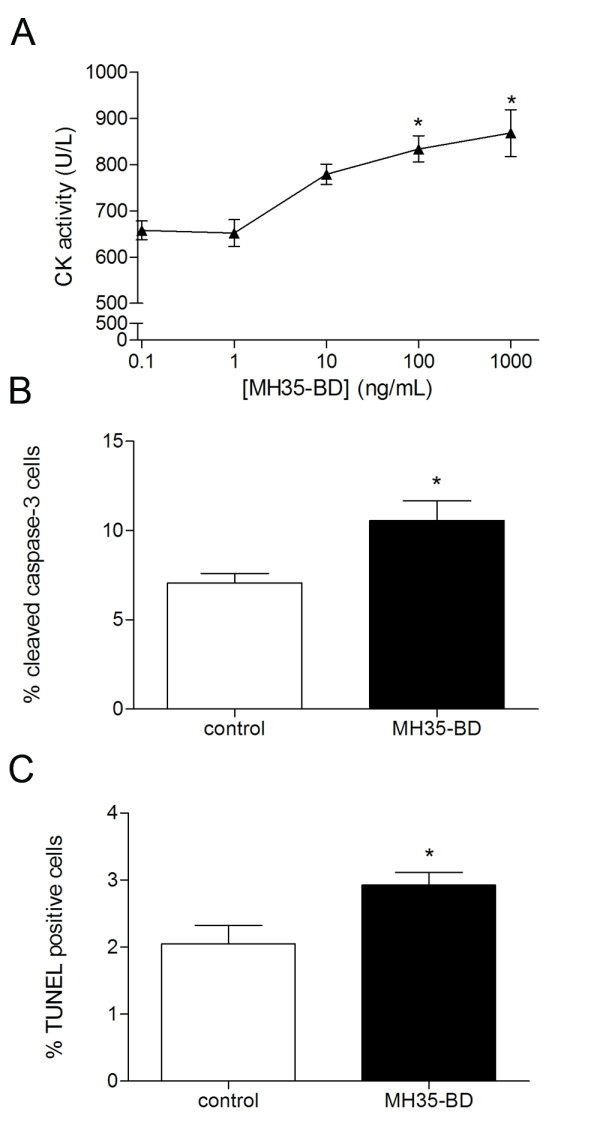
**MH35-BD blocks the biological effect of LIF in growth media showing increased differentiation and apoptotic signalling**. (A) Creatine kinase activity of myoblasts after 24 hours of differentiation with increasing concentrations of MH35-BD; 100 ng/mL and 1000 ng/mL MH35-BD significantly increased creatine kinase activity. Asterisk (*) indicates *P *< 0.05 with one-way ANOVA and Dunnet's post-hoc test for all concentrations compared to control (n = 4). (B) The percentage of cells positive for cleaved caspase-3 after 24 hours differentiation was increased with MH35-BD (100 ng/mL) treatment. Asterisk (*) indicates *P *< 0.05 with Student's t-test compared to control (n = 4). (C) The percentage of cells positive for TUNEL after 24 hours of differentiation was increased with MH35-BD (100 ng/mL) treatment. Asterisk (*) indicates *P *< 0.05 with Student's t-test compared to control (n = 4).

To determine if bovine LIF was affecting the differentiation of myoblasts we employed the LIFR antagonist MH35-BD, a mutant form of LIF that binds the LIFR but does not elicit signalling to any great degree and is therefore a competitive antagonist of LIF [21]. Myoblasts were incubated with different concentrations of MH35-BD in GM for the culture period 24 hours before differentiation, then induced to differentiate with DM, and differentiation and differentiation associated apoptosis examined. CK activity at t = 24 was significantly increased compared to control with MH35-BD exposure at concentrations of 100 ng/mL and greater (Figure 6A), suggesting that inhibition of bovine LIF promoted differentiation. Similarly at t = 24 the percentage of active caspase-3 and TUNEL positive cells was significantly increased with 100 ng/mL MH35-BD (Figure 6B and 6C). Overall inhibition of LIF present in growth media with 100 ng/mL MH35-BD, presumably bovine LIF, increased CK activity and the percentage of active caspase-3 and TUNEL positive cells by 27%, 34% and 30%, respectively, above control levels. This suggests that bovine LIF present in fetal bovine serum can indeed elicit effects through the murine LIF receptor. We also investigated the LIF receptor components LIFR and gp130. Transcripts for LIFR decreased sharply following differentiation while gp130 remained relatively consistently expressed throughout (Figure 5).

## Discussion

Given the numerous reports of the effect of LIF on myoblasts [11,13,14,19,22,23] it is not surprising that transcripts for LIFR and gp130 were detected in myoblasts in this study as they would be required for LIF to elicit an effect. However it was interesting that LIFR transcripts decreased after the induction of differentiation suggesting desensitization to LIF stimulation in the more differentiated cultures.

We could detect no endogenous LIF production by myoblasts secreted into conditioned media; however, we did find evidence for the presence of bovine LIF in growth media containing fetal bovine serum. Given that bovine LIF possesses 77% amino acid sequence homology to murine LIF and murine LIF is routinely used to improve development of cultured bovine embryos [24,25], indicating that the bovine LIF receptor is sensitive to murine LIF, it would be believable that the murine LIF receptor is sensitive to bovine LIF. In our study addition of the competitive LIFR antagonist MH35-BD increased measures of differentiation and differentiation associated apoptosis, the opposite effect of stimulation with exogenous murine LIF, suggesting that bovine LIF present in GM was inhibiting differentiation of myoblasts and this was inhibited by MH35-BD. Therefore we hypothesize that the long held method for inducing differentiation *in vitro *by switching from the growth factor rich fetal bovine serum to low growth factor normal horse serum may act to induce differentiation at least in part by removal of LIF as an inhibitor of myogenic differentiation and as one of many possible growth factors present in relatively high levels in fetal bovine serum.

Although changes in transcripts for LIF were observed during differentiation we could not detect any endogenously produced LIF secreted into conditioned media even when transcripts were highest, suggesting that relatively little LIF is produced by myoblasts under these conditions. Consistent with this is that relatively large concentrations (10 ng/mL) of exogenous LIF as well as an unknown quantity of bovine LIF in growth media are required to achieve a maximum effect of LIF inhibiting differentiation, suggesting that the LIFR is under-saturated by any endogenous LIF produced by myoblasts. If myoblasts do not produce significant amounts of LIF under these conditions then it might insinuate that without stimuli additional to what is provided in this study, myoblasts may not be the major source of LIF *in vivo *observed to be up-regulated in muscle injury [10] and might be due to other cells such as macrophages, fibroblasts or neurons present in the muscle tissue. A recent study utilizing a more sensitive ELISA method for the detection of LIF has suggested that cultured myotubes can produce approximately 3.5 pg/mL of LIF into media, compared to approximately 0.8 pg/mL found in the non-conditioned horse serum containing differentiation media control [26]. This amount is approximately 1,000 fold less than is typically found to have an effect on cells and would therefore support our supposition that myogenic cultures do not produce significant amounts of LIF.

Earlier studies examining the effect of LIF on myogenic differentiation suggested that LIF neither promoted nor inhibited differentiation [23], but more recently it was demonstrated that LIF inhibits myogenic differentiation [11,22]. The present study confirms the most recent reports that LIF does indeed inhibit myogenic differentiation and provides a greater understanding of the mechanisms underlying this effect and additional insight into the involvement of LIF in differentiation-associated apoptosis as well as the regulation of LIF and receptor components during myogenesis. PI3K signalling promotes myogenic differentiation [27] and mediates LIF dependent inhibition of apoptosis in myoblasts [14]. In the present study inhibition of the PI3K pathway significantly reduced fusion index but did not prevent LIF-dependent decreases in caspase-3 activation indicating that this inhibition of caspase-3 activation was not PI3K mediated. LIF appeared to inhibit differentiation and caspase-3 activation by a MEK and extracellular signal-regulated kinase (ERK) dependent mechanism. An example of how the MEK/ERK pathway might influence caspase-3 activity is demonstrated by ERK phosphorylation of caspase-9 at Thr-125, which inhibits caspase-3 cleavage and activation [28]. Therefore we may speculate that activation of MEK by LIF and subsequent inhibition of caspase-3 activation may be directly responsible for the inhibition of differentiation observed. Consistent with the observation that knockout of caspase-3 in myoblasts inhibits myogenic differentiation [8], we observed that the reversible caspase-3 inhibitor Ac-DEVD-CHO inhibited myogenic differentiation. We also observed active caspase-3 positive myoblasts that did not show classical apoptotic morphology which supports the notion that caspase-3 is associated with myogenic differentiation and not only apoptosis.

Inhibition of caspase-3 activity with Ac-DEVD-CHO achieved the same effect as LIF with inhibition of myotube formation and CK activity reduced by a very similar amount. Interestingly the combination of LIF and Ac-DEVD-CHO did not further decrease caspase-3 or CK activity compared to either treatment alone. One might have expected that if LIF were to only inhibit the cleavage of caspase-3 in 50% of cells (possibly due to ERK phosphorylation of caspase-9) as seen with immunocytochemical analysis, the remaining cells that possessed active caspase-3 would still have high caspase-3 proteolytic activity that could be further inhibited. Treatment with Ac-DEVD-CHO however did not achieve this, which could suggest that LIF regulates proteins that inhibit not just the cleavage of caspase-3 but also proteolysis at the active site such as X-linked inhibitor of apoptosis protein (XIAP) that is shown to be up-regulated with LIF treatment compared to without LIF treatment in apoptotic cells [14]. It could also suggest that inhibition of caspase-3 activity with Ac-DEVD-CHO had reached a plateau, that is caspase-3 activity was inhibited to a maximum amount achievable. Nevertheless the combination of immunocytochemical and enzymatic assays strongly support the proposition that LIF inhibits caspase-3 activation and proteolytic activity that is normally increased with myogenic differentiation and that this correlates with inhibition of myogenic differentiation.

Inhibition of caspase pathways via dominant negative expression of death receptors DR5 and FADD decreases myoD messenger [29] and may explain why LIF stimulation was also accompanied by decreased levels of myoD messenger in our study. Expression of c-fos is also negatively associated with the transcriptional activity of myoD and myogenin inhibiting CK transcription by these transcription factors [18]. This agrees with our finding that LIF up-regulates expression of the c-fos transcripts and also decreases CK enzyme activity and inhibits differentiation. As p21 expression is dependent on myoD expression [5] it is not surprising that LIF exposure also down-regulated p21 mRNA. Corresponding to this decrease in cdk inhibitor p21, LIF exposure maintained myoblasts in a proliferative state for a longer period of time despite culture conditions promoting cell cycle withdrawal and differentiation. Altogether this suggests that LIF inhibits myogenic differentiation by multiple mechanisms that include inhibition of caspase-3 activation possibly leading to a decrease in levels of myoD and myogenin, up-regulation of c-fos, which may lead to inhibition of the transcriptional activity of myoD and myogenin, and down-regulation of p21 leading to delayed cell cycle withdrawal. In addition to activation of caspase-3, LIF also inhibited DNA fragmentation in populations of differentiating cells. Although DNA fragmentation is classically associated with apoptosis it is also demonstrated that caspase-induced DNA strand breaks can be important for the regulation of differentiation. Caspase-activated DNAse (CAD) can be activated by caspase-3 and allows for up-regulation of p21 to promote myogenic differentiation [30]. Therefore the reduced DNA fragmentation observed with LIF treatment may reflect the down-regulation of p21 and inhibition of differentiation observed. DNA fragmentation within myogenic cells can occur *in vivo *and macrophage influx into damaged muscle is temporally correlated with decreased DNA fragmentation in myogenic cells [31]. As LIF is a cytokine potentially secreted by macrophages in damaged muscle [32] it could be suggested that LIF is but one such secreted factor that may mediate this DNA fragmentation in regenerating muscle tissue.

## Conclusions

This study demonstrates for the first time the mechanisms involved in inhibition of myogenic differentiation by LIF, in particular the importance of caspase-3 inhibition as a mediator of inhibition of differentiation by LIF. Although little is known about the effect of LIF on necrosis of muscle fibers, inflammation, innervation and other factors important to skeletal muscle regeneration, this study indicates a role for LIF on the myogenic cells required for muscle regeneration, which may be to prevent precocious differentiation.

## Methods

### Cell culture and reagents

C2C12 myoblast cells were obtained from Amercian Type Culture Collection (ATCC; Manassas, VA, USA). Recombinant murine LIF was purchased from Millipore (Billerica, MA, USA). All reagents were obtained from Sigma-Aldrich (Castle Hill, NSW, Australia) unless otherwise stated. For all experiments comparing LIF-treated to -untreated myoblasts, myoblasts were seeded such that 24 hours prior to induction of differentiation they were approximately 40-50% of total plate density and were incubated with 10 ng/mL LIF in growth media (10% fetal bovine serum in DMEM) or growth media alone for 24 hours. Following this, the cells, which were approximately 90% - 100% of total plate density, were washed with PBS and differentiation media (2% horse serum in DMEM) was added. Differentiation media was replenished every 24 hours until the end point of the experiment.

### Protein expression and purification

The gene encoding the LIF mutant MH35-BD was subcloned and expressed as a glutathione-S-transferase (GST) fusion protein in *Escherichia coli*, strain BL21 using the Overnight Express Autoinduction System (Novagen). After 24 hours of incubation, the cells were harvested by centrifuging at 10,000 rpm for 10 minutes at 4°C and the pellet was stored at -20°C. Later, the pellet (approximately 40 g) was resuspended in 40 mL of freshly made buffer (50 mM Tris-HCl pH 7.4, 500 mM NaCl, 1 mM Ethylene diaminetetra acetatic acid, 1 mM Dithiothreitol, 1X protease inhibitor cocktail) and lysed by sonication. The cell debris was then pelleted by centrifugation at 25,000 rpm for 15 min at 4°C and the supernatant was filtered through a 0.44 micron filter. The filtered supernatant was then passed through a glutathione-agarose (Sigma-Aldrich, Castle Hill, NSW, Australia) chromatography column twice. Following this, the column was washed with 5 column volumes of the same buffer and incubated with 25 units of Thrombin (5 U of thrombin/mg of protein) for 2 days at room temperature (16 to 25°C). Thrombin was added in order to cleave the protein from GST. For final elution, the GST column was attached in series with a benzamidine column, which traps thrombin thereby separating it from the purified protein in the eluate. The purity of the protein was confirmed by SDS-PAGE and silver staining.

### MH35-BD bioactivity assay

To determine the biological activity of MH35-BD, a cell proliferation assay employing Ba/F3-hLIF-R/hgp130 cells was used. This cell line originates from an IL-3-dependent cell line derived from murine pro B lymphocytes, which do not normally express LIF-R or gpl30 [33]. To generate the stable Ba/F3-hLIF-R/hgp130 cell line, Ba/F3 cells were stably transfected with the human LIF-R (hLIF-R) and human gp130 (hgp130), after which they proliferated rapidly in the presence of hLIF [34]. Their proliferation is markedly decreased in the presence of hLIF antagonists. The assay procedure was conducted as described previously [35].

### Creatine kinase activity assay

C2C12s were cultured in 24-well plates until the end point of the experiment. Cultures were then washed twice with phosphate buffered saline (PBS) and lysed with 150 μL lysis buffer containing 40 mM MES buffer (2-(N-morpholino)ethanesulfonic acid), 50 mM Trizma base, 1% (v/v) Triton X-100 and complete protease inhibitor cocktail (1 tablet per 50 mL lysis buffer obtained from Roche Applied Science (Castle Hill, NSW, Australia)). Insoluble material was pelleted by centrifugation and the resulting supernatant was used for analysis of creatine kinase (CK) activity. The supernatant (10 μL) was added in triplicate for each sample to a 96-well plate followed by 200 μL of CK-NAC (Thermo Scientific, Scoresby, VIC, Australia). The plate was then run on a PARADIGM MicroPlate Detection Platform (Beckman Coulter, Gladesville, NSW, Australia) set to 37°C and the change in absorbance at 340 nm over 3 minutes (20 second intervals) measured. The change in absorbance was converted to activity in units per liter (U/L) by multiplication by the following factor k.

Where TV is the total reaction volume (0.210 mL), A is the millimolar absorption coefficient of NADH at 340 nM (6.3), SV is the sample volume (0.010 mL) and P is the pathlength of light (determined to be 0.533 cm by spectrophotometric comparison of absorbances with known pathlengths).

### Caspase-3 colormetric proteolytic activity assay

All reagents for assessing caspase-3 activity including Ac-DEVD-CHO were obtained from the Caspase-3 Assay Kit, Colormetric (Sigma Aldrich). C2C12s were cultured in 12-well plates and treated as described in 'Cell culture and reagents'. At the end point of the experiment cells were washed and lysed in 100 μL of lysis buffer from the kit and 80 μL of the sample was added to 10 μL of the 10× assay buffer and 10 μL of the 2 mM Ac-DEVD-pNA chromogenic substrate into 96-well plates. They were incubated for 10 hours at 37°C after which the absorbance of the plates at 405 nm was read. The absorbance was divided by the time in minutes incubated in order to achieve the change absorbance units per minute. CK activity was also determined as described above using 10 μL of the lysates.

### Antibodies

Cleaved Caspase-3 (Asp175) (5A1E) Rabbit monoclonal antibody was obtained from Cell Signalling Technology (Danvers, MA, USA). Monoclonal Anti-Myosin MY-32 (Skeletal, Fast) antibody produced in mouse was purchased from Sigma-Aldrich (Castle Hill, NSW, Australia). Mouse anti-LIF (clone 2H2.2) was purchased from Millipore (Billerica, MA, USA). Fluorescent secondary antibodies donkey anti-rabbit AlexaFluor488, goat anti-mouse AlexaFluor594 and streptavidin AlexaFluor594 were purchased from Invitrogen (Carlsbad, CA, USA). The secondary horseradish peroxidise conjugated anti-mouse was purchased from Cell Signalling Technology (Danvers, MA, USA).

### Fluorescent immunocytochemistry

For dual labelling of caspase-3 and myosin heavy chain (MYHC), cultures were fixed by adding a volume of 4% paraformaldehyde (PFA) equal to the volume of media present and incubated for 15 minutes. Following washing with PBS, cultures were blocked with a solution containing 5% (v/v) donkey serum, 5% (v/v) goat serum and 0.3% Triton X-100 in PBS for 60 minutes at room temperature (RT). They were then incubated with 1/200 dilutions of rabbit anti-cleaved caspase-3 and mouse anti-MYHC in PBS overnight at 4°C. After washing with PBS, 1:250 dilutions of the secondary antibodies donkey anti-rabbit AlexFluor488 and goat anti-mouse AlexaFluor 594 in PBS were added to the cultures and incubated for 45 minutes at RT. They were then counterstained with 4',6-diamidino-2-phenylindole (DAPI) and mounted on slides for imaging.

For dual labelling of bromodeoxyuridine (BrdU) incorporated cells and desmin BrdU cell proliferation, ELISA kit (Roche Applied Science, Castle Hill, NSW, Australia) was utilized following the recommended procedure up until antibody incubations. Then, cultures were incubated with the mouse anti-BrdU and rabbit anti-desmin antibodies for 90 minutes at RT. They were washed and then incubated with goat anti-mouse AlexaFluor594 and donkey anti-rabbit AlexaFluor488 for 45 minutes at RT. They were counterstained with DAPI and mounted on slides.

Slides were imaged on a Leica TCS SP2 confocal setup (Leica Microsystems, Wetzlar, Germany) using photomultiplier tubes for absorption and emission spectra of DAPI, AlexaFluor488 and AlexaFluor594 and imaged sequentially to avoid bleed-through from overlapping spectra. Digital photographic images were taken and counted. From MYHC and desmin staining, fusion indexes were calculated as the proportion of nuclei contained within syncytia (2 or more nuclei per cytoplasm) compared to the total nuclei present within a field of view. BrdU and active caspase-3 positive cells were calculated as the percentage of positive compared to total nuclei present in a field of view. A minimum of 1,000 nuclei from multiple fields of view were counted for all replicates and the total of all fields of view calculated as a percentage for that particular sample.

### TUNEL staining

Immunofluorescent labelling of DNA fragmentation in myoblasts was achieved using the DeadEnd™ Fluoremetric TUNEL system from Promega (Madison, WI, USA). In short, myoblasts were fixed with 4% PFA, permeabilised in PBS with 0.3% Triton X-100, and incubated with fluorescein-12-dUTP and Terminal Deoxynucleotidyl Transferase for 1 hour at 37°C. The reaction was stopped and excess non-incorporated fluorescein-12-dUTP washed off and cultures counterstained with DAPI before being mounted on slides and visualized for fluorescent microscopy.

### Western immunoblotting

For detection of LIF in conditioned media from cultures as well as non-conditioned media, samples were incubated at 95°C for 5 minutes with an equal volume of a reducing gel loading buffer containing 100 mM Tris pH 6.8, 2% (w/v) SDS, 5% (v/v) β- mercaptoethanol, 15% (v/v) glycerol and 0.1% (w/v) bromophenol blue. The samples and molecular weight marker SeeBlue^® ^Plus2 (Invitrogen, Carslbad, CA, USA) were then resolved on a 12% (w/v) Tris buffered polyacrylamide gel with a constant current of 30 mA for approximately 2 hours. The resolved protein was transferred onto BioTrace™PVDF transfer membrane (Pall Corporation, Melbourne, VIC, Australia) and blocked with 5% (w/v) skim milk in PBS with 0.1% Tween-20 overnight at 4°C. They were then incubated with 1:500 dilution of mouse anti-LIF antibody overnight at 4°C. The horseradish peroxidise conjugated anti-mouse antibody was used as a secondary and incubated for 1 hour at RT and then washed off. The membrane was then incubated with Amersham ECL™ Western Blotting detection reagents and exposed with Amersham Hyperfilm ECL film, which were both purchased from GE Healthcare Life Science (Rydalmere, NSW, Australia).

### Quantitative PCR assay

Total RNA was extracted from cultures grown in 6-well plates using the SV Total RNA Isolation System (Promega, Madison, WI, USA). RNA (1 μg) from each sample was used for reverse transcription with M-MLV Reverse Transcriptase (Promega, Madison, WI, USA). The cDNA synthesised was subjected to real-time quantitative PCR using SYBR Green qPCR Supermix UDG (Invitrogen, Carlsbad, CA, USA) on a Roche LightCycler 480 real-time PCR unit. The derived cycle threshold (Ct) values were analyzed using the relative expression software tool (REST) [36] in order to determine statistical difference. Data were expressed as the mean of normalized expression in order to display linear levels of transcripts of interest normalized to the housekeeper cyclophilin A, which was the most adequate housekeeper as it possessed the least variability across all samples compared to other housekeepers tested including HPRT, GAPDH and β-actin using the "Bestkeeper" excel based tool [37].

Where A is the efficiency of amplification of the housekeeper, B is the efficiency of the gene of interest, × is the housekeeper Ct and y is the gene of interest Ct. Oligonucleotide sequences for all genes assessed are shown in Table 1.

**Table 1:** List of oligonucleotide sequences used for real-time qPCR

Gene name	Forward (5'-3')	Reverse (5'-3')
c-fos	atgggctctcctgtcaacac	tgtcaccgtggggataaagt

cyclophilinA	ggccgatgacgagccc	tgtctttggaactttgtctgcaa

gp130	catagtcgtgcctgtgtgct	gtgaccactgggcaatatga

LIF	caagaatcaactggcacagc	agtggggttcaggaccttct

LIFR	agaagaactggctcccattg	ggatgtcgtcccatttcact

MyoD	tacagtggcgactcagatgc	tagtaggcggtgtcgtagcc

Myogenin	agtgaatgcaactcccacag	acgatggacgtaagggagtg

p21	gcagaccagcctgacagattc	ttcagggttttctcttgcagaag

## List of abbreviations

BP: blocking peptide; CAD: caspase-activated DNAse; CDK: cyclin dependent kinase; CK: creatine kinase; CM: conditioned medium; DM: differentiating medium; DR5: death receptor 5; EGF: epidermal growth factor; ERK: extracellular signal-regulated kinase; FADD: Fas-associated protein with death domain; FGF: fibroblast growth factor; GM: growth medium; LIF: leukemia inhibitory factor; LIFR: leukemia inhibitor factor receptor; MRF: muscle regulatory factor; MYHC: myosin heavy chain; MEK: mitogen activated protein kinase and extracellular signal regulated kinase kinase;PI3K: phosphotidylinositol-3 kinase; NCM: non-conditioned medium; TA: tibialis anterior; TUNEL: terminal deoxynucleotidyl transferase mediated dUTP Nick End Labeling; XIAP: X-linked inhibitor of apoptosis protein

## Competing interests

The authors declare that they have no competing interests.

## Authors' contributions

LH carried out cell culture experiments and drafted the manuscript. AU expressed and purified MH35-BD recombinant protein. JJ participated in MH35-BD expression/purification and assisted in manuscript editing. ET assisted in experimental design and manuscript editing. JDW assisted in experimental design and manuscript editing. All authors have read and approved the final manuscript.
